# 4,4-Difluoro-1,3,5,7-tetra­methyl-4-bora-3a,4a-diaza-*s*-indacene

**DOI:** 10.1107/S1600536808024057

**Published:** 2008-08-06

**Authors:** Hai-Jun Xu

**Affiliations:** a Ordered Matter Science Research Center, College of Chemistry and Chemical Engineering, Southeast University, Nanjing 210096, People’s Republic of China

## Abstract

In the title compound, C_13_H_15_BF_2_N_2_, the two pyrrole rings are almost coplanar, with a dihedral angle of 3.08 (10)°. The BF_2_ plane is almost perpendicular to the boron–dipyrromethene ring plane, with a dihedral angle of 89.99 (7)°.

## Related literature

For related literature, see: Bergström *et al.* (2002 ▶); Kollmannsberger *et al.* (1998 ▶); Kuhn *et al.* (1990 ▶); Trieflinger *et al.* (2005 ▶).
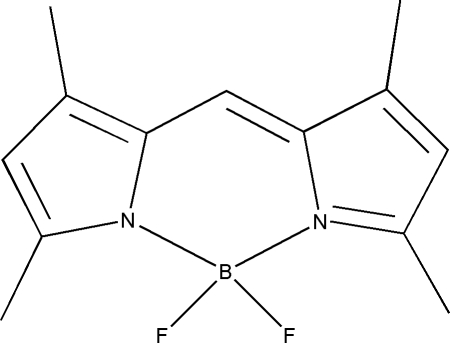


## Experimental

### 

#### Crystal data

C_13_H_15_BF_2_N_2_
*M* *_r_* = 248.08
Monoclinic, 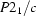

*a* = 7.6909 (8) Å
*b* = 14.3392 (15) Å
*c* = 11.8334 (10) Å
β = 111.108 (5)°
*V* = 1217.4 (2) Å^3^
*Z* = 4
Mo *K*α radiation
μ = 0.10 mm^−1^
*T* = 293 (2) K
0.30 × 0.20 × 0.20 mm

#### Data collection

Rigaku Mercury2 diffractometer
Absorption correction: multi-scan (*CrystalClear*; Rigaku, 2005 ▶) *T*_min_ = 0.961, *T*_max_ = 0.974
6540 measured reflections
2396 independent reflections
1963 reflections with *I* > 2σ(*I*)
*R*_int_ = 0.019

#### Refinement

*R*[*F* ^2^ > 2σ(*F* ^2^)] = 0.040
*wR*(*F* ^2^) = 0.126
*S* = 1.06
2396 reflections
167 parameters
H-atom parameters constrained
Δρ_max_ = 0.27 e Å^−3^
Δρ_min_ = −0.24 e Å^−3^

### 

Data collection: *CrystalClear* (Rigaku, 2005 ▶); cell refinement: *CrystalClear*; data reduction: *CrystalClear*; program(s) used to solve structure: *SHELXS97* (Sheldrick, 2008 ▶); program(s) used to refine structure: *SHELXL97* (Sheldrick, 2008 ▶); molecular graphics: *SHELXTL* (Sheldrick, 2008 ▶); software used to prepare material for publication: *SHELXL97*.

## Supplementary Material

Crystal structure: contains datablocks I, global. DOI: 10.1107/S1600536808024057/at2574sup1.cif

Structure factors: contains datablocks I. DOI: 10.1107/S1600536808024057/at2574Isup2.hkl

Additional supplementary materials:  crystallographic information; 3D view; checkCIF report

## Figures and Tables

**Fig. 1.**
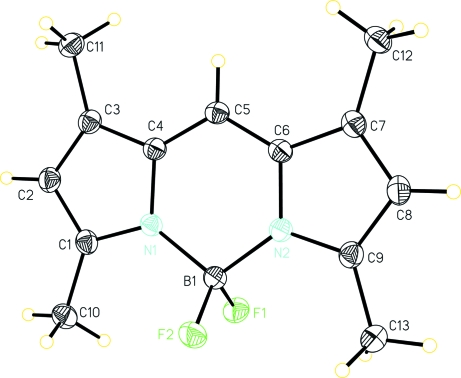
A view of the title compound with the atomic numbering scheme. Displacement ellipsoids are drawn at the 30% probability level.

## supplementary crystallographic information

### Comment

Boron-dipyrromethene (BODIPY) dyes are excellent fluorophores, with a high molar extinction coefficient and high fluorescence quantum yield, which have recently received considerable attention with regard to the design of fluorescence labels and biomolecular sensors (Bergström *et al.*, 2002; Trieflinger *et al.*, 2005). We hope to synthesize the boron-dipyrromethene (BODIPY) dyes containing phenanthroline group by the use of the reaction of 1,10-phenanthroline-2,9-dicarbaldehyde with 2,4-dimethyl-1*H*-pyrrole, however we only obtained the title compound (I) unexpectedly.

As shown in Fig. 1, the BODIPY skeleton is formed by three conjugated heterocyclic rings which is nearly coplanar. The C1/C2/C3/C4/N1 and C6/C7/C8/C9/N2 rings make dihedral angles of 1.37 (8) ° and 2.37 (9) °, respectively, with the N1/C4/C5/C6/N2/B1 ring plane. The average bond lengths for B—N and B—F and the average N—B—N and F—B—F and F—B—N angles indicate a tetrahedral BF_2_N_2_ configuration and are in good agreement with previous published data (Kuhn, *et al.*, 1990). The F1/B1/F2 plane is almost perpendicular to the BODIPY ring plane [dihedral angle = 89.99 (0.07) °].

### Experimental

Compound (I) was prepared in one-pot reaction (Kollmannsberger *et al.*, 1998). Pyrrole (4 mmol) and 1,10-phenanthroline-2,9-dicarbaldehyde (1 mmol) were dissolved in newly dry CH_2_Cl_2_ (80 ml) under argon atmosphere. One drop of trifluoroacetic acid was added and the solution was stirred at room temperature until thin layer chromatography showed complete consumption of the aldehyde. At this point, a solution of dichlorodicyanobenzoquinone (DDQ, 2 mmol) in dry CH_2_Cl_2_ (20 ml) was added, and the mixture was stirred for additional 15 min. The reaction mixture was then treated with triethylamine (3 ml) and boron trifluoride etherate (3 ml). After stirring for another 30 min, the dark brown solution was washed with water (3×50 ml) and brine (50 ml), dried over Na_2_SO_4_, and concentrated at reduced pressure. The crude product was purified by silica-gel flash column chromatography and recrystallization from CHCl_3_/hexane. ^1^H NMR (300 MHz, CDCl_3_): δ = 7.37(s, 1 H), 6.13 (s, 2 H), 2.43 (s, 6 H), 2.16 (s, 6 H). Esi-Mass: 229.47 [M—F]^+^. Single crystals of (I) were obtain from a hexane-chloroform solution.

### Refinement

Positional parameters of all the H atoms were calculated geometrically with C—H = 0.93 - 0.96 Å and were allowed to ride on the C atoms to which they are bonded, with *U*_iso_(H) = 1.2 or 1.5U_eq_(C).

### Crystal data

**Table:**

C_13_H_15_B_1_F_2_N_2_	*F*_000_ = 520
*M**_r_* = 248.08	*D*_x_ = 1.353 Mg m^−^^3^
Monoclinic, *P*2_1_/*c*	Mo *K*α radiation λ = 0.71073 Å
Hall symbol: -P 2ybc	Cell parameters from 2682 reflections
*a* = 7.6909 (8) Å	θ = 2.8–27.9º
*b* = 14.3392 (15) Å	µ = 0.10 mm^−^^1^
*c* = 11.8334 (10) Å	*T* = 293 (2) K
β = 111.108 (5)º	Block, red
*V* = 1217.4 (2) Å^3^	0.30 × 0.20 × 0.20 mm
*Z* = 4	

### Data collection

**Table:**

Rigaku Mercury2 diffractometer	2396 independent reflections
Radiation source: fine-focus sealed tube	1963 reflections with *I* > 2σ(*I*)
Monochromator: graphite	*R*_int_ = 0.019
Detector resolution: 13.6612 pixels mm^-1^	θ_max_ = 26.0º
*T* = 293(2) K	θ_min_ = 2.3º
φ and ω scans	*h* = −8→9
Absorption correction: empirical (using intensity measurements)(CrystalClear; Rigaku, 2005)	*k* = −17→16
*T*_min_ = 0.961, *T*_max_ = 0.974	*l* = −14→12
6540 measured reflections	

### Refinement

**Table:**

Refinement on *F*^2^	Secondary atom site location: difference Fourier map
Least-squares matrix: full	Hydrogen site location: inferred from neighbouring sites
*R*[*F*^2^ > 2σ(*F*^2^)] = 0.040	H-atom parameters constrained
*wR*(*F*^2^) = 0.126	*w* = 1/[σ^2^(*F*_o_^2^) + (0.0669*P*)^2^ + 0.3386*P*] where *P* = (*F*_o_^2^ + 2*F*_c_^2^)/3
*S* = 1.06	(Δ/σ)_max_ = 0.007
2396 reflections	Δρ_max_ = 0.27 e Å^−^^3^
167 parameters	Δρ_min_ = −0.24 e Å^−^^3^
Primary atom site location: structure-invariant direct methods	Extinction correction: none

### Special details

**Table:**

Geometry. All e.s.d.'s (except the e.s.d. in the dihedral angle between two l.s. planes) are estimated using the full covariance matrix. The cell e.s.d.'s are taken into account individually in the estimation of e.s.d.'s in distances, angles and torsion angles; correlations between e.s.d.'s in cell parameters are only used when they are defined by crystal symmetry. An approximate (isotropic) treatment of cell e.s.d.'s is used for estimating e.s.d.'s involving l.s. planes.
Refinement. Refinement of *F*^2^ against ALL reflections. The weighted *R*-factor *wR* and goodness of fit *S* are based on *F*^2^, conventional *R*-factors *R* are based on *F*, with *F* set to zero for negative *F*^2^. The threshold expression of *F*^2^ > σ(*F*^2^) is used only for calculating *R*-factors(gt) *etc*. and is not relevant to the choice of reflections for refinement. *R*-factors based on *F*^2^ are statistically about twice as large as those based on *F*, and *R*- factors based on ALL data will be even larger.

### Fractional atomic coordinates and isotropic or equivalent isotropic displacement parameters (Å^2^)

**Table:**

	*x*	*y*	*z*	*U*_iso_*/*U*_eq_	
F1	0.90473 (14)	−0.13462 (7)	0.77523 (9)	0.0416 (3)	
F2	0.59217 (14)	−0.11875 (7)	0.68465 (9)	0.0429 (3)	
N1	0.77362 (17)	0.01985 (9)	0.76358 (11)	0.0296 (3)	
N2	0.72797 (18)	−0.10054 (9)	0.90212 (12)	0.0302 (3)	
C1	0.7917 (2)	0.06479 (12)	0.66762 (15)	0.0333 (4)	
C2	0.8203 (2)	0.15979 (12)	0.69475 (16)	0.0368 (4)	
H2A	0.8371	0.2054	0.6438	0.044*	
C3	0.8194 (2)	0.17455 (11)	0.80964 (15)	0.0331 (4)	
C4	0.7899 (2)	0.08638 (11)	0.85312 (14)	0.0296 (4)	
C5	0.7747 (2)	0.06086 (11)	0.96176 (14)	0.0292 (4)	
H5A	0.7865	0.1065	1.0199	0.035*	
C6	0.7427 (2)	−0.03013 (11)	0.98692 (14)	0.0296 (4)	
C7	0.7146 (2)	−0.06999 (12)	1.08848 (15)	0.0331 (4)	
C8	0.6836 (2)	−0.16395 (12)	1.06306 (16)	0.0377 (4)	
H8A	0.6607	−0.2083	1.1133	0.045*	
C9	0.6926 (2)	−0.18101 (11)	0.94874 (15)	0.0343 (4)	
C10	0.7815 (3)	0.01588 (13)	0.55463 (16)	0.0419 (4)	
H10A	0.8497	−0.0416	0.5749	0.063*	
H10B	0.8346	0.0547	0.5093	0.063*	
H10C	0.6536	0.0030	0.5067	0.063*	
C11	0.8416 (3)	0.26491 (12)	0.87641 (17)	0.0411 (4)	
H11A	0.9168	0.3066	0.8498	0.062*	
H11B	0.9011	0.2539	0.9618	0.062*	
H11C	0.7213	0.2923	0.8606	0.062*	
C12	0.7199 (2)	−0.01832 (13)	1.19915 (16)	0.0388 (4)	
H12A	0.6385	−0.0482	1.2334	0.058*	
H12B	0.6797	0.0448	1.1779	0.058*	
H12C	0.8449	−0.0183	1.2574	0.058*	
C13	0.6716 (3)	−0.27203 (12)	0.88435 (17)	0.0426 (4)	
H13A	0.7826	−0.2851	0.8680	0.064*	
H13B	0.5669	−0.2690	0.8094	0.064*	
H13C	0.6516	−0.3206	0.9342	0.064*	
B1	0.7490 (2)	−0.08597 (13)	0.77801 (16)	0.0310 (4)	

### Atomic displacement parameters (Å^2^)

**Table:**

	*U*^11^	*U*^22^	*U*^33^	*U*^12^	*U*^13^	*U*^23^
F1	0.0484 (6)	0.0363 (6)	0.0467 (6)	0.0106 (4)	0.0251 (5)	−0.0002 (4)
F2	0.0463 (6)	0.0438 (6)	0.0326 (6)	−0.0101 (4)	0.0070 (5)	−0.0031 (4)
N1	0.0304 (7)	0.0317 (7)	0.0272 (7)	0.0027 (5)	0.0109 (6)	0.0002 (5)
N2	0.0303 (7)	0.0297 (7)	0.0298 (7)	0.0031 (5)	0.0099 (6)	0.0009 (5)
C1	0.0331 (8)	0.0369 (9)	0.0312 (9)	0.0055 (7)	0.0132 (7)	0.0039 (7)
C2	0.0432 (9)	0.0346 (9)	0.0366 (9)	0.0045 (7)	0.0192 (8)	0.0060 (7)
C3	0.0321 (8)	0.0319 (8)	0.0371 (9)	0.0031 (6)	0.0145 (7)	0.0018 (7)
C4	0.0271 (8)	0.0311 (8)	0.0299 (8)	0.0035 (6)	0.0093 (6)	−0.0009 (6)
C5	0.0257 (7)	0.0311 (8)	0.0301 (8)	0.0031 (6)	0.0094 (6)	−0.0023 (6)
C6	0.0270 (7)	0.0337 (8)	0.0278 (8)	0.0046 (6)	0.0095 (6)	−0.0005 (6)
C7	0.0296 (8)	0.0383 (9)	0.0312 (9)	0.0046 (7)	0.0108 (7)	0.0036 (7)
C8	0.0418 (9)	0.0367 (9)	0.0364 (9)	0.0023 (7)	0.0163 (8)	0.0081 (7)
C9	0.0343 (9)	0.0325 (9)	0.0346 (9)	0.0023 (6)	0.0104 (7)	0.0030 (7)
C10	0.0510 (11)	0.0448 (10)	0.0336 (9)	0.0035 (8)	0.0197 (8)	−0.0003 (7)
C11	0.0495 (10)	0.0319 (9)	0.0447 (10)	−0.0003 (8)	0.0204 (9)	−0.0010 (7)
C12	0.0411 (9)	0.0460 (10)	0.0318 (9)	0.0039 (8)	0.0163 (8)	0.0005 (7)
C13	0.0511 (11)	0.0327 (9)	0.0421 (10)	−0.0013 (8)	0.0146 (8)	0.0008 (7)
B1	0.0326 (9)	0.0312 (9)	0.0287 (9)	0.0011 (7)	0.0105 (7)	−0.0004 (7)

### Geometric parameters (Å, °)

**Table:**

F1—B1	1.397 (2)	C7—C8	1.382 (2)
F2—B1	1.392 (2)	C7—C12	1.492 (2)
N1—C1	1.356 (2)	C8—C9	1.401 (2)
N1—C4	1.3974 (19)	C8—H8A	0.9300
N1—B1	1.546 (2)	C9—C13	1.490 (2)
N2—C9	1.348 (2)	C10—H10A	0.9600
N2—C6	1.399 (2)	C10—H10B	0.9600
N2—B1	1.548 (2)	C10—H10C	0.9600
C1—C2	1.399 (2)	C11—H11A	0.9600
C1—C10	1.487 (2)	C11—H11B	0.9600
C2—C3	1.378 (2)	C11—H11C	0.9600
C2—H2A	0.9300	C12—H12A	0.9600
C3—C4	1.414 (2)	C12—H12B	0.9600
C3—C11	1.495 (2)	C12—H12C	0.9600
C4—C5	1.382 (2)	C13—H13A	0.9600
C5—C6	1.380 (2)	C13—H13B	0.9600
C5—H5A	0.9300	C13—H13C	0.9600
C6—C7	1.416 (2)		
			
C1—N1—C4	107.60 (14)	C8—C9—C13	127.78 (15)
C1—N1—B1	127.78 (14)	C1—C10—H10A	109.5
C4—N1—B1	124.57 (13)	C1—C10—H10B	109.5
C9—N2—C6	107.47 (13)	H10A—C10—H10B	109.5
C9—N2—B1	127.51 (13)	C1—C10—H10C	109.5
C6—N2—B1	125.02 (13)	H10A—C10—H10C	109.5
N1—C1—C2	109.00 (14)	H10B—C10—H10C	109.5
N1—C1—C10	122.75 (15)	C3—C11—H11A	109.5
C2—C1—C10	128.25 (15)	C3—C11—H11B	109.5
C3—C2—C1	108.66 (14)	H11A—C11—H11B	109.5
C3—C2—H2A	125.7	C3—C11—H11C	109.5
C1—C2—H2A	125.7	H11A—C11—H11C	109.5
C2—C3—C4	106.26 (14)	H11B—C11—H11C	109.5
C2—C3—C11	127.90 (15)	C7—C12—H12A	109.5
C4—C3—C11	125.84 (15)	C7—C12—H12B	109.5
C5—C4—N1	120.68 (14)	H12A—C12—H12B	109.5
C5—C4—C3	130.84 (15)	C7—C12—H12C	109.5
N1—C4—C3	108.48 (14)	H12A—C12—H12C	109.5
C6—C5—C4	122.19 (14)	H12B—C12—H12C	109.5
C6—C5—H5A	118.9	C9—C13—H13A	109.5
C4—C5—H5A	118.9	C9—C13—H13B	109.5
C5—C6—N2	120.14 (14)	H13A—C13—H13B	109.5
C5—C6—C7	131.18 (15)	C9—C13—H13C	109.5
N2—C6—C7	108.66 (14)	H13A—C13—H13C	109.5
C8—C7—C6	106.01 (14)	H13B—C13—H13C	109.5
C8—C7—C12	128.52 (15)	F2—B1—F1	108.55 (14)
C6—C7—C12	125.47 (16)	F2—B1—N1	110.45 (13)
C7—C8—C9	108.40 (15)	F1—B1—N1	110.32 (13)
C7—C8—H8A	125.8	F2—B1—N2	110.53 (13)
C9—C8—H8A	125.8	F1—B1—N2	109.77 (13)
N2—C9—C8	109.46 (14)	N1—B1—N2	107.21 (13)
N2—C9—C13	122.76 (15)		
